# Changes in family situation and concurrent changes in working life: a 15-year longitudinal analysis

**DOI:** 10.1136/fmch-2023-002438

**Published:** 2024-04-04

**Authors:** Mo Wang, Pia Svedberg, Jurgita Narusyte, Annina Ropponen

**Affiliations:** 1 Department of Clinical Neuroscience, Division of Insurance Medicine, Karolinska Institutet, Stockholm, Sweden; 2 Finnish Institute of Occupational Health, Helsinki, Finland

**Keywords:** epidemiology, family planning policy, family planning services, occupational health

## Abstract

**Objective:**

Currently, little is known regarding changes in family situation with concurrent changes in working life. This study aimed to examine whether changes in family situation (based on living with children and/or marrying/divorcing) were associated with changes in working life and whether the associations were influenced by sex, genetics and early life environment.

**Design:**

Prospective cohort study.

**Setting and participants:**

Data from Swedish national registers of 16 410 twins were used. Fixed-effects logistic regression models assessing ORs with 95% CIs were applied to examine associations between changes in family situation and working life controlling for time-invariant effects and adjusted for covariates, and conditional models to account for confounding of genetics and early life environment.

**Results:**

Changes in individuals life situation from being single and living without children to married and living with children were associated with transitioning from unsustainable (ie, having unemployment or sickness absence/disability pension) to sustainable working life (men: OR 2.40, 95% CI 2.26 to 2.56; women: OR 1.68, 95% CI 1.59 to 1.78). Changes from being married to single, in contrast, attenuated the likelihood of transitioning to a sustainable working life. Moreover, changes in men’s working life seem to be more dependent on changes in family situation compared with women. Genetic factors and early life environment play a role in the associations.

**Conclusions:**

Family formation increases the likelihood of a more stable working life whereas divorce is a risk factor for work interruptions. Our study emphasises that family formation improves the work life situation and to a higher degree for men.

**WHAT IS ALREADY KNOWN ON THIS TOPIC**

- Changes in the family situation, such as marriage, the birth of a child or divorce, have implications for an individual’s health and participation in paid work in terms of unemployment or absences from work.

**WHAT THIS STUDY ADDS**

- Studies are few with longitudinal design to investigate changes in family situations and participation in paid work. Investigation of twins adds knowledge of the role of genetic factors and early life environment on the longitudinal associations between family situation and participation in paid work.

**HOW THIS STUDY MIGHT AFFECT RESEARCH, PRACTICE OR POLICY**

- Family formation and remaining in a family could be promoted to support a sustainable working life or transition towards a sustainable working life. However, different strategies for women and men should be considered.

## Introduction

Family life plays an important role in determining well-being and shaping decisions about working life.^1 2^ Also, major changes in the family situation, such as marriage, the birth of a child, which can be considered as positive events, or from the negative perspective, divorce, have implications for an individual’s health and help to structure the human life course.^3–5^ Focusing on the positive aspects of family situation that can be retrieved from national registers of family formation, previous research has found that individuals living with a partner and being in paid work are associated with better health outcomes when measured in terms of morbidity and mortality.^6 7^ Marriage is deemed to offer a direct form of social and financial support and it can reduce the risk of unhealthy behaviours, such as poor diet or alcohol use.^8^ The marriage rate in Sweden is comparatively low within Europe but increasing, at 2.3/1000 persons. Sweden also has a relatively high divorce rate (roughly the same as marriages), which places Sweden in the top three countries in divorce rates in Europe.^9 10^ However, living with someone without marriage is very high in Sweden and even among young adults.^10^ This highlights the family situation that could provide financial independence, social support, social recognition and self-esteem, which could also have a positive influence on health.^11^ Moreover, various and sometimes even negative factors are associated with family situation and participation in paid work including sex, age, musculoskeletal pain and mental disorders.^12 13^ Although the association between family situation, working life and subjective well-being is well documented,^14 15^ there are still questions left unanswered. Importantly, many of the studies of family situation and participation in paid work have been cross-sectional,^16 17^ whereas few studies have been longitudinal investigating how changes in family situation impact concurrent changes in participation in paid work.^18 19^ By following individuals prospectively for a long time, we would get a better understanding of how individuals move through different positive and negative family and work participation situations throughout life.

Participation in paid work among women has increased in most Western countries, but women still show lower rates of participation in paid work than men and still do a large proportion of the household chores.^20 21^ Previous studies have shown that women are more likely to decrease their working hours after a change occurred in the family, for example, the birth of the first child while men’s participation in paid work remains relatively stable.^20 22^ Women are also more often granted sickness absence or disability pension benefits than men,^19 23^ which can be assessed as adverse effect for participation in paid work. Hence, it can be hypothesised that changes in family situation may have a stronger impact on participation in paid work for women than men. On the other hand, multiple roles, such as combining paid work and family, are likely to be beneficial to women’s health, as multiple roles deliver social support, financial independence and self-esteem.^24 25^ However, little attention has been paid to sex differences on the association between changes in family situation and sustainable participation in paid work. In welfare countries such as in the Nordic countries, not being in paid work due to life events such as family formation (having children), sickness absence, disability pension or unemployment are economically compensated via available and relevant social benefits adding up to a decent income level.^26 27^ This might affect interruptions from paid work while also influencing high participation of women in paid work. In addition, in Sweden, more women (24%) work part time than men (9%).^28^ Taken together, it is important to consider whether changes in family situation affect concurrent changes in working life also among men.

Furthermore, there is a lack of longitudinal research based on data from twins to investigate the role of genetic factors and early life environment (such as shared family environment mainly in childhood for twins reared together) on the associations between family situation and working life. Previous twin studies have suggested that genetic factors play a role for both interruptions in working life and family situation because they account for 36%–50% of the variance in sickness absence and disability pension and approximately 27% for family-related life events.^29–31^ In addition, a twin study is a powerful tool to account for unmeasured confounders, since co-twins are matched on genetic (100% for monozygotic (MZ) and on average 50% for dizygotic (DZ) twin pairs) and common rearing environmental variation (100% for both MZ and DZ twins).^32^

In this study, sustainable working life is defined as living and working conditions that support people in engaging and remaining in work throughout an extended working life and is deemed to bring deeper insights on changes in working life over the life course.^33^ We will measure sustainable working life as ‘not having or having very little work incapacity that is sickness absence or disability pension, or other interruptions such as unemployment’ as has been done before based on the detailed Swedish register data.^33 34^ The aims of this study were to elucidate (1) whether changes in family situation (based on living with children and/or marrying/cohabitating vs divorcing) were associated with concurrent changes in working life (based on degree of sustainable working life) over a 15-year period while accounting for genetic factors and early life environment and (2) possible sex differences in these associations.

## Methods

### Sample and data

This prospective twin cohort study was based on data from the Swedish Twin project Of Disability pension and Sickness absence. Information on exposure, covariates and outcomes was obtained from the following national registers and merged by using the unique personal identification numbers which are provided to all Swedish residents:

1. The Swedish Social Insurance Agency: MicroData for Analyses of Social insurance register dates for all sickness absence (>2 weeks) and disability pension from 1994 onwards.
2. Statistics Sweden: the Longitudinal Integration Database for Health Insurance and Labour Market Studies Register for family situation, unemployment, old-age pension and sociodemographic variables from 1990 onwards.^35^
3. The National Board of Health and Welfare: Data on date of death were obtained from the Cause of Death Register from 1961 onwards and data on the diagnosed diseases were collected from the Swedish National Patient Register (date and diagnosis for inpatient and specialised outpatient care starting from 1987 and from 2001, respectively).

**Figure 1:**
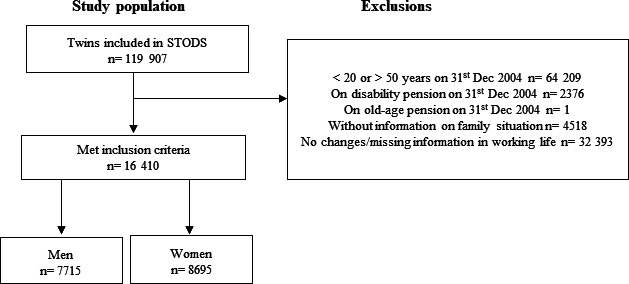
Flow chart for the study population. STODS, Swedish Twin project Of Disability pension and Sickness absence.

The inclusion criteria were (1) information available on family situation between 2005 and 2020 and (2) being 20–50 years old and not on old-age or disability pension on 31 December 2004. The exclusion criteria for the fixed-effects analysis were no changes or missing information (eg, due to death) of sustainable working life during 2005–2020. The final cohort consisted of 16 410 twins. Among them, 3441 were complete twin pairs whereof 2190 were MZ twins, 1586 same-sex DZ twins, 1862 opposite-sex DZ twins and 1244 were of unknown zygosity. There were 388 male twin pairs and 477 female twin pairs discordant for working life in 2005, that is, one twin in a pair had sustainable working life and the other twin did not. The sample was then stratified by sex (figure 1). The MZ twins sharing 100% of genes are optimally matched by default, whereas same-sexed DZ twins share 50% of their segregation genes while being matched for age and sex. Limiting analyses for them and studying men and women separately provides control for these aspects while assessing potential sex differences.

### Social insurance system in Sweden

All people in Sweden above the age of 16 years are eligible for sickness absence benefits if having income from work or unemployment or parental benefits. Sickness absence benefits amount up to 80% of lost income if unable to work due to a disease or injury. Employees received sick pay for the first 2 weeks of the sick leave spell from the employer, thereafter from the Social Insurance Agency. All other groups had benefits from the Social Insurance Agency after one qualifying day, except for self-employed, who could have more qualifying days. All residents with a permanently impaired work capacity due to disease or injury can be granted disability pension, which covers up to approximately 65% of lost income. Unemployment benefits can be granted to unemployed individuals with a defined minimum income from work. The individuals need to be registered as unemployed at the Swedish Public Employment Service to show their ability and willingness to take a job right away to receive compensation from an unemployment insurance fund. In addition to social insurance, many Swedish workers are covered by collective labour agreements. However, these agreements do not affect social insurance, but minimum wages and other conditions related to employment.^36^

### Exposure

Exposure was measured as changes in family situation, which was based on living with children and/or marrying or cohabitating/divorcing. However, for parsimony in text and especially tables, ‘married’ will be used for marriage or cohabitation. Family situation was measured in each year between 2005 and 2020 with the following categories: (1) married and living without children; (2) married and living with children; (3) single and living without children and (4) single and living with children. Married status included living with partner and cohabitant whereas single included divorced, separated or widowed. The categories of changes in family situation during 2005–2020 are described in online supplemental table 1.

10.1136/fmch-2023-002438.supp1 Supplementary data

### Outcomes

The cohort was followed from 2005 to 2020 regarding changes in working life, that is, from unsustainable working life to sustainable working life. Sustainable working life was measured by using the information on main labour market status in each year of follow-up as has been done before.^37 38^ The labour market status included sickness absence and disability pension (>180 days with sickness benefits or disability pension); unemployment (>180 days with unemployment benefits); old-age pension (more than half of yearly income from old-age pension) or employment (ie, in paid work and did not fulfil the criteria sickness absence, disability pension, unemployment or old-age pension). Individuals were considered to have sustainable working life if employed in any follow-up year. The changes in working life were considered from unsustainable working life (reference group; ie, having unemployment >180 days or sickness absence/disability pension >180 days or more than half of yearly income from old-age pension) to sustainable working life (ie, in paid work and did not have unemployment >180 days, nor sickness absence/disability pension >180 days, nor more than half of yearly income from old-age pension) between 2005 and 2020.

### Covariates

Covariates from baseline consisted of age (continuous variable), levels of education (ie, highest level of completed education, classified into elementary (<10 years of education (reference group), medium (10–12 years in education) and higher education (>12 years in education)), type of living area (big cities, medium-sized cities, small cities/villages (reference group)), and mental and somatic diagnoses according to the main diagnosis (mental diagnoses in each year between 2005 and 2020 were based on the International Classification of Diseases, 10th edition (ICD-10): F00–F99 while somatic diagnoses were based on ICD-10 codes other than F00–F99) from inpatient and specialised outpatient care (table 1).

**Table 1:** Frequencies of sociodemographic factors, zygosity, diagnoses, family situation and working life in 2005 among total participants and sex subcohorts

Variables	Total N=16 410	Men N=7715	Women N=8695	P value
	N (%)	N (%)	N (%)	
**Age (mean, SD**)	35.4 (9.5)	35.7 (9.7)	35.2 (9.4)	0.002
Zygosity				<0.001
Monozygotic	4254 (25.9%)	1781 (23.1%)	2473 (28.4%)	
Dizygotic same sex	3958 (24.1%)	1787 (23.2%)	2171 (25.0%)	
Dizygotic opposite sex	3067 (18.7%)	1744 (22.6%)	1323 (15.2%)	
Unknown zygosity	5131 (31.3%)	2403 (31.1%)	2728 (31.4%)	
Education (years)				<0.001
<10	2023 (12.3%)	1156 (15.0%)	867 (10.0%)	
10–12	9108 (55.5%)	4355 (56.4%)	4753 (54.7%)	
>12	5279 (32.2%)	2204 (28.6%)	3075 (35.4%)	
Type of living area*				0.042
Big cities	6046 (36.8%)	2779 (36.0%)	3267 (37.6%)	
Medium-sized cities	5956 (36.3%)	2800 (36.3%)	3156 (36.3%)	
Small cities/villages	4408 (26.9%)	2136 (27.7%)	2272 (26.1%)	
Mental diagnoses				
Mental diagnoses from outpatient care	341 (2.1%)	131 (1.7%)	210 (2.4%)	0.001
Mental diagnoses from inpatient care	106 (0.6%)	49 (0.6%)	57 (0.7%)	0.871
Somatic diagnoses				
Somatic diagnoses from outpatient care	5267 (32.1%)	2001 (25.9%)	3266 (37.6%)	<0.001
Somatic diagnoses from inpatient care	1173 (7.1%)	322 (4.2%)	851 (9.8%)	<0.001
Family situation				<0.001
Married† without children	1221 (7.4%)	486 (6.3%)	735 (8.5%)	
Married† with children	5309 (32.4%)	2145 (27.8%)	3164 (36.4%)	
Single‡ without children	8714 (53.1%)	4890 (63.4%)	3824 (44.0%)	
Single‡ with children	1166 (7.1%)	194 (2.5%)	972 (11.2%)	
Working life				0.357
Unsustainable working life	4922 (30.0%)	2341 (30.3%)	2581 (29.7%)	
Sustainable working life	11 488 (70.0%)	5374 (69.7%)	6114 (70.3%)	

*Type of living area: big cities: Stockholm, Göteborg and Malmö; medium-sized cities: cities with more than 90 000 inhabitants within 30 km distance from the centre of the city; small cities/villages.

†Married includes living with partner; cohabitant.

‡Single includes divorced, separated or widowed.

### Statistical analyses

Statistical differences in the distributions of covariates, exposures and outcomes were assessed with χ^2^ and t-tests. P values less than 0.05 indicate a significant difference.

First, the changes in family situation associated with concurrent changes in working life were determined by using fixed-effects logistic regression in the whole sample stratified by sex.^39^ This longitudinal approach can remove any known or unknown confounding effects from individual differences that do not change over time (eg, sex) by design. This approach uses an analytical sample with different values for the dependent variable, that is, sustainable working life, on at least two measurement occasions.^40^ The ORs with 95% CIs from the fixed-effects approach reflect the association between the exposure category (vs the reference category) and a transition from unsustainable working life to sustainable working life. Besides the crude model, the multivariate model was adjusted for age, education, type of living area, mental and somatic diagnoses measured in each year between 2005 and 2020. Thus, using the fixed-effects approach with our 15-year follow-up with measures from every year, enabled us to model only within-subject variation and control for the time-invariant characteristics.

**Figure 2:**
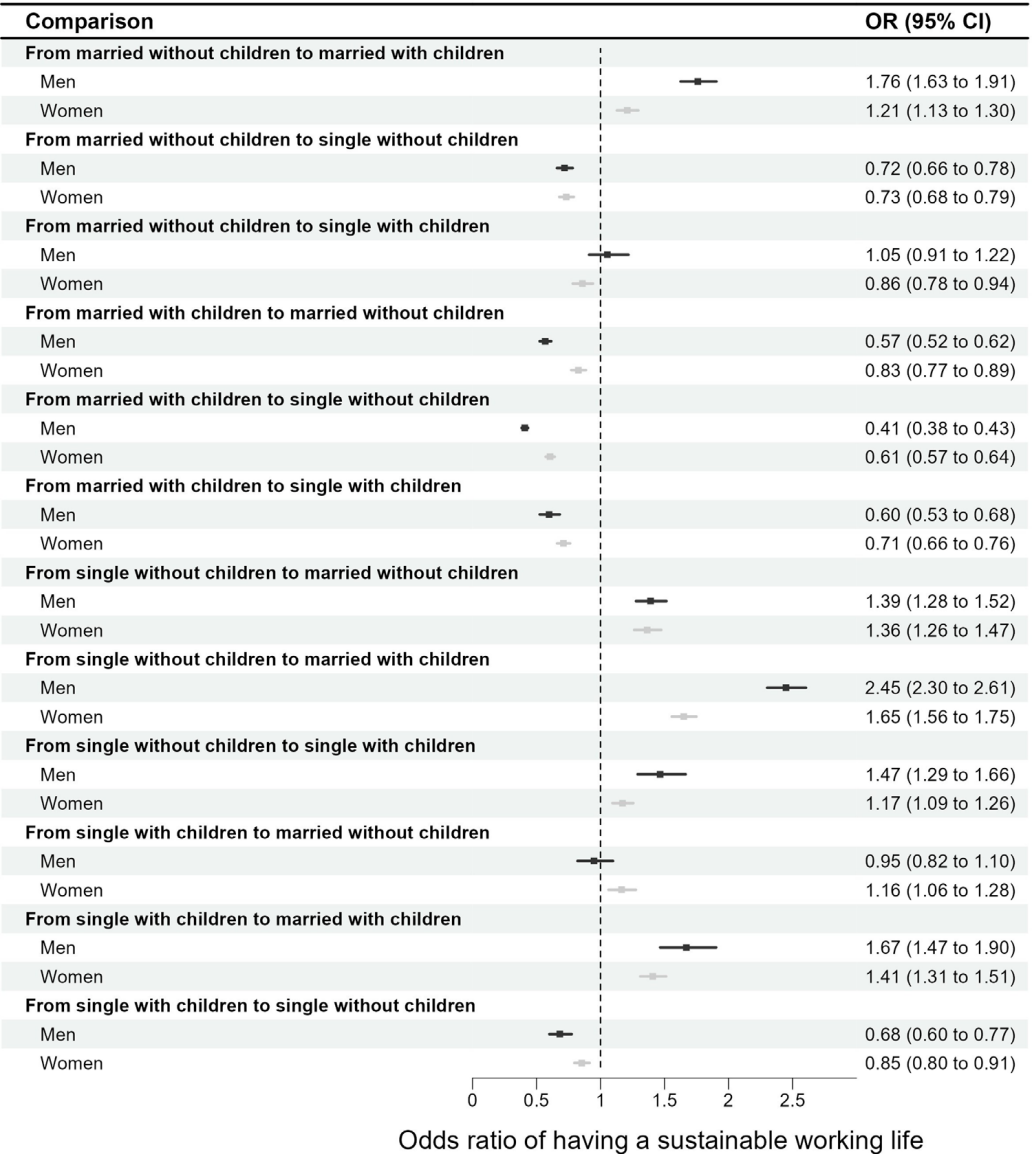
Forest plot of ORs with 95% CI based on the average partial effects to compare associations of changes in family situation and a sustainable working life between men and women.

Co-twin analyses were performed separately for sexes on outcome discordant twin pairs (ie, one twin in a pair had sustainable working life in 2005 and the other twin did not) using conditional logistic regression models. As twins are matched on genetics (100% for MZ twins and on average 50% for DZ twins) and shared environment (100% for both MZ and DZ) while growing up (if raised together), the co-twin analysis adjusts for these factors. If the association found in the age-adjusted and sex-adjusted analyses of the whole sample disappears or becomes weaker in the analyses of discordant twin pairs there is a suggestion that genetic factors and early life environment are influencing the association. If an association is still observed after controlling for genetic factors and early life environment this would instead suggest a direct link between the exposure and the outcome.^41^ The analyses were based on discordant twin pairs (ie, 388 male twin pairs and 477 female twin pairs) for the outcome where one twin in a pair had sustainable working life in 2005 and the twin sibling did not. All analyses were conducted by SAS Statistical Software V.9.4. Both for the whole sample and for the discordant twins, average partial effects,^42^ were estimated to add interpretation of the results (see online supplemental tables S2 and S3). To visualise the comparison between men and women, we used the OR of the adjusted model (model 1) for a forest plot (figure 2).

## Results

### Descriptive statistics

Frequencies of sociodemographic factors, zygosity, diagnoses, family situation and working life in 2005 in the whole sample and for men and women are presented in table 1. In the whole sample, there were 8695 women (53%) and the average age was approximately 35 years old (SD=9.5). The majority of the participants (70.0%) had sustainable working life in 2005. Among the sex subcohorts, a large proportion of women had a higher educational level (35.4% with more than 12 years of education vs 28.6% for men) and a somatic diagnosis (37.6% from specialised outpatient care vs 25.9% for men and 9.8% from inpatient care vs 4.2% for men) and were more likely to be married (44.9% vs 34.1% for men) and single living with children (11.2% vs 2.5% for men) than men.

### Family situation changes and working life

Table 2 presents associations between changes of family situation and working life in men in the whole sample and of the co-twins discordant for outcome. In the whole sample, all the changes in family situations were associated with a statistically significant odds of transitioning from unsustainable to sustainable working life in the crude model (range of ORs 0.42–2.37). In model 1, we adjusted for sociodemographic factors including age, education and type of living area and the ORs for the transitions showed some changes, indicating influence from sociodemographic factors on the associations. The increased odds of transitioning from unsustainable to sustainable working life remained even after additional control for mental and somatic diagnoses, particularly for single men living without children who changed to married living with children (OR 2.40, 95% CI 2.26 to 2.56). We also observed decreased odds of transitioning from unsustainable to sustainable working life in, for example, change from being married living with children to single living without children (OR 0.42, 95% CI 0.39 to 0.44). In the analyses of discordant twin pairs, the ORs attenuated and became non-significant for most of the changes in family situation. On the other hand, the increased ORs remained significant for men who changed from single living without children to married living with/without children and single men living with children who changed to married living with children in the co-twin analysis among MZ twin pairs. Also, married men who became single still showed reduced odds of transitioning from unsustainable to sustainable working life among MZ twin pairs.

**Table 2:** The fixed-effects analysis on associations between changes in family situation and transitions from unsustainable working life to sustainable working life among men analysed between 2005 and 2020 in the whole sample (n=7715) and of the co-twins discordant for outcome (n=776)

Changes in family situation	Whole sample			Discordant twin pairs		
	Crude model	Model 1*	Model 2†	Conditional all	DZ‡ pairs	MZ§ pairs
	OR (95% CI)	OR (95% CI)	OR (95% CI)	OR (95% CI)	OR (95% CI)	OR (95% CI)
From married¶ without children to married¶ with children**	**2.05 (1.90 to 2.21**)	**1.76 (1.63 to 1.91**)	**1.75 (1.61 to 1.89**)	1.30 (1.00 to 1.69)	0.94 (0.61 to 1.44)	1.18 (0.74 to 1.86)
From married¶ without children to single†† without children**	**0.86 (0.80 to 0.94**)	**0.72 (0.66 to 0.78**)	**0.73 (0.67 to 0.79**)	**0.52 (0.40 to 0.69**)	0.71 (0.45 to 1.13)	**0.35 (0.21 to 0.57**)
From married¶ without children to single†† with children**	**1.25 (1.08 to 1.44**)	1.05 (0.91 to 1.22)	1.07 (0.93 to 1.24)	**0.51 (0.32 to 0.82**)	1.03 (0.43 to 2.45)	0.46 (0.20 to 1.04)
From married¶ with children to married¶ without children‡‡	**0.49 (0.45 to 0.53**)	**0.57 (0.52 to 0.62**)	**0.57 (0.53 to 0.62**)	0.77 (0.59 to 1.00)	1.07 (0.70 to 1.64)	0.85 (0.54 to 1.35)
From married¶ with children to single†† without children‡‡	**0.42 (0.40 to 0.45**)	**0.41 (0.38 to 0.43**)	**0.42 (0.39 to 0.44**)	**0.40 (0.34 to 0.48**)	0.76 (0.53 to 1.09)	**0.30 (0.22 to 0.40**)
From married¶ with children to single†† with children‡‡	**0.61 (0.54 to 0.69**)	**0.60 (0.53 to 0.68**)	**0.61 (0.54 to 0.70**)	**0.40 (0.26 to 0.59**)	1.10 (0.50 to 2.42)	**0.39 (0.19 to 0.80**)
From single†† without children to married¶ without children§§	**1.16 (1.07 to 1.26**)	**1.39 (1.28 to 1.52**)	**1.37 (1.26 to 1.50**)	**1.91 (1.44 to 2.53**)	1.40 (0.88 to 2.23)	**2.88 (1.76 to 4.72**)
From single†† without children to married¶ with children§§	**2.37 (2.23 to 2.52**)	**2.45 (2.30 to 2.61**)	**2.40 (2.26 to 2.56**)	**2.48 (2.07 to 2.97**)	1.31 (0.92 to 1.87)	**3.38 (2.52 to 4.53**)
From single†† without children to single†† with children§§	**1.44 (1.27 to 1.64**)	**1.47 (1.29 to 1.66**)	**1.48 (1.30 to 1.68**)	0.98 (0.66 to 1.46)	1.44 (0.65 to 3.21)	1.32 (0.66 to 2.65)
From single†† with children to married¶ without children¶¶	**0.80 (0.70 to 0.93**)	0.95 (0.82 to 1.10)	0.93 (0.81 to 1.08)	**1.94 (1.22 to 3.10**)	0.97 (0.41 to 2.31)	2.18 (0.96 to 4.95)
From single†† with children to married¶ with children¶¶	**1.64 (1.44 to 1.87**)	**1.67 (1.47 to 1.90**)	**1.63 (1.43 to 1.86**)	**2.53 (1.68 to 3.80**)	0.91 (0.41 to 1.99)	**2.56 (1.25 to 5.26**)
From single†† with children to single†† without children¶¶	**0.69 (0.61 to 0.79**)	**0.68 (0.60 to 0.77**)	**0.68 (0.60 to 0.77**)	1.02 (0.69 to 1.51)	0.69 (0.31 to 1.54)	0.76 (0.38 to 1.52)

Statistically significant OR with 95%CI are presented in bold.

*Model 1: adjusted for age, education and type of living area between 2005 and 2020.

†Model 2: adjusted for age, education and type of living area, mental and somatic diagnoses between 2005 and 2020.

‡DZ: Dizygotic twins.

§MZ: Monozygotic twins.

¶Married includes living with partner; cohabitant.

**Reference group: married without children.

††Single includes divorced, separated or widowed.

‡‡Reference group: married with children.

§§Reference group: single without children.

¶¶Reference group: single with children.

We found similar associations between changes in family situation and transitions from unsustainable to sustainable working life between 2005 and 2020 among women (table 3). Particularly, women who changed from being single living without children to married living with children had higher odds of transitioning from unsustainable to sustainable working life (OR 1.68, 95% CI 1.59 to 1.78) whereas married women living with children who changed to single living without children had significantly decreased odds (OR 0.59, 95% CI 0.56 to 0.63) in the whole sample. These ORs even remained significant in the analyses of discordant twins, indicating a direct (ie, not affected by genetic factors and early life environment) link between changes in family situation and concurrent changes in working life. The comparisons between men and women based on the ORs are shown in figure 2.

**Table 3:** The fixed-effects analysis on associations between changes in family situation and transitions from unsustainable working life to sustainable working life among women analysed between 2005 and 2020 in the whole sample (n=8695) and of the co-twins discordant for outcome (n=954)

Changes in family situation	Whole sample			Discordant twin pairs		
	Crude model	Model 1*	Model 2†	Conditional all	DZ‡ pairs	MZ§ pairs
	OR (95% CI)	OR (95% CI)	OR (95% CI)	OR (95% CI)	OR (95% CI)	OR (95% CI)
From married¶ without children to married¶ with children**	**1.40 (1.31 to 1.49**)	**1.21 (1.13 to 1.30**)	**1.24 (1.16 to 1.33**)	0.87 (0.70 to 1.07)	**0.67 (0.48 to 0.94**)	1.06 (0.78 to 1.46)
From married¶ without children to single†† without children**	**0.83 (0.77 to 0.90**)	**0.73 (0.68 to 0.79**)	**0.74 (0.68 to 0.80**)	**0.43 (0.34 to 0.55**)	**0.39 (0.26 to 0.57**)	**0.50 (0.36 to 0.70**)
From married¶ without children to single†† with children**	1.01 (0.93 to 1.11)	**0.86 (0.78 to 0.94**)	**0.87 (0.79 to 0.96**)	**0.51 (0.39 to 0.68**)	**0.48 (0.30 to 0.79**)	**0.64 (0.43 to 0.94**)
From married¶ with children to married¶ without children‡‡	**0.72 (0.67 to 0.77**)	**0.83 (0.77 to 0.89**)	**0.80 (0.75 to 0.86**)	1.15 (0.93 to 1.43)	**1.49 (1.06 to 2.10**)	0.94 (0.69 to 1.29)
From married¶ with children to single†† without children‡‡	**0.60 (0.56 to 0.63**)	**0.61 (0.57 to 0.64**)	**0.59 (0.56 to 0.63**)	**0.50 (0.43 to 0.59**)	**0.58 (0.43 to 0.78**)	**0.47 (0.38 to 0.59**)
From married¶ with children to single†† with children‡‡	**0.73 (0.68 to 0.78**)	**0.71 (0.66 to 0.76**)	**0.70 (0.65 to 0.75**)	**0.59 (0.49 to 0.72**)	0.72 (0.48 to 1.07)	**0.60 (0.46 to 0.79**)
From single†† without children to married¶ without children§§	**1.20 (1.11 to 1.29**)	**1.36 (1.26 to 1.47**)	**1.35 (1.25 to 1.46**)	**2.30 (1.82 to 2.92**)	**2.58 (1.74 to 3.82**)	**2.00 (1.42 to 2.81**)
From single†† without children to married¶ with children§§	**1.67 (1.58 to 1.77**)	**1.65 (1.56 to 1.75**)	**1.68 (1.59 to 1.78**)	**1.99 (1.70 to 2.34**)	**1.73 (1.28 to 2.34**)	**2.13 (1.70 to 2.66**)
From single†† without children to single†† with children§§	**1.22 (1.13 to 1.30**)	**1.17 (1.09 to 1.26**)	**1.18 (1.10 to 1.27**)	1.18 (0.97 to 1.44)	1.25 (0.84 to 1.84)	1.28 (0.97 to 1.69)
From single†† with children to married¶ without children¶¶	0.99 (0.90 to 1.08)	**1.16 (1.06 to 1.28**)	**1.15 (1.05 to 1.26**)	**1.94 (1.48 to 2.56**)	**2.07 (1.26 to 3.38**)	**1.56 (1.06 to 2.30**)
From single†† with children to married¶ with children¶¶	**1.38 (1.28 to 1.48**)	**1.41 (1.31 to 1.51**)	**1.43 (1.33 to 1.53**)	**1.68 (1.38 to 2.06**)	1.39 (0.93 to 2.07)	**1.66 (1.26 to 2.18**)
From single†† with children to single†† without children¶¶	**0.82 (0.77 to 0.88**)	**0.85 (0.80 to 0.91**)	**0.85 (0.79 to 0.91**)	0.84 (0.69 to 1.03)	0.80 (0.54 to 1.19)	0.78 (0.59 to 1.03)

Statistically significant OR with 95%CI are presented in bold.

*Model 1: adjusted for age, education and type of living area between 2005 and 2020.

†Model 2: adjusted for age, education and type of living area, mental and somatic diagnoses between 2005 and 2020.

‡DZ: Dizygotic twins.

§MZ: Monozygotic twins.

¶Married includes living with partner; cohabitant.

**Reference group: married without children.

††Single includes divorced, separated. or widowed.

‡‡Reference group: married with children.

§§eference group: single without children.

¶¶Reference group: single with children.

Regarding sex differences, the ORs for changes from being single living without children to single living with children or to married living with children as well as changes from being married living without children to married living with children in men (table 2) were higher than women (table 3) in the multivariate model in the whole sample. In contrast, married men living with children who changed to married living without children or to single living without children and single men living with children who changed to single living without children showed significantly lower ORs of transitioning from unsustainable to sustainable working life in comparison to women. The ORs were similar in magnitude and direction for both sexes (ie, no sex differences) in the analyses of discordant twin pairs.

## Discussion

This study of over 16 000 Swedish twins sought to examine the association of changes in family situation and concurrent changes in working life over a 15-year period, and whether the association was influenced by genetic factors and early life environment as well as sex. The sustainable working life, defined as paid work and not having a long-term period of unemployment, sickness absence and disability pension was applied as the measure for changes in working life. The findings highlight several different changes in family situation based on living with children and marrying and divorcing as important to change in working life. Specifically, individuals who changed from single living without children to married living with children were associated with transitioning from unsustainable to sustainable working life. On the other hand, changes from being married to single reduced the possibility of transitioning from unsustainable to sustainable working life. There was also some support of confounding for family situations and working life in relation to genetic factors and early life environment and across sex. Results suggest that the impact from changes in family situation on changes in working life is different between men and women and genetic factors and early life environment play a role in the associations.

### Changes in family situation and labour market changes

The association between transitioning to a family situation such as establishing a family with children and having a sustainable working life aligns with previous research, reporting that individuals who have a partner tend to have a more optimal health and healthier lifestyle than those who live alone.^4 6 43 44^ It might be interpreted by the beneficial effects of the partnership including the economic contributions from the spouse or cohabitant, sharing of knowledge regarding healthy behaviour and being an important source of support in case of illness.^45^ Our findings further suggest that the changes in family situation have implications for an individual’s labour market changes. It is likely that family formation (eg, single individuals who get married and start to live with children) may impact health, emotional, social and financial support and consequently working careers.^46^ In contrast to having the family, our results revealed that individuals who become separated, divorced and widowed as well as single individuals who do not anymore live with children are less likely to transition to sustainable working life. Compared with married individuals, being divorced and living without children may be associated with health disadvantages, as those individuals may experience increased stress from the negative changes in family situation and lack sources of support from family.^8^ Individuals with poorer health are more likely to have unsustainable working life according to the health selection hypothesis, such as being unemployed, on sickness absence or disability pension.^47^

### Twins to clarify associations

We further observed that the significant results in the associations between some changes in family situation and working life changes in the whole sample disappeared in the analyses of discordant twin pairs, indicating an influence from genetic factors and early life environment. This result is in line with an earlier study based on the Finnish twin cohort, showing the role of genetic factors and early life environment on the associations between the number of family-related life events and disability pension.^48^ Since sickness absence, disability pension and life events have a genetic component,^29–31^ we assumed that genetic factors and early life environment would play a role in the associations of changes in family situation and working life. However, some associations were still significant even after controlling for genetic factors and early life environment, for example, among single individuals living without children who changed to married and married individuals living with children who changed to single. This finding suggests a direct relationship (independent from genetic factors and early life environment) between those changes in the family situation and changes in working life and thereby emphasising that family formation and remaining a relationship might be important for a better working life. Still, the current knowledge on the role of genetic factors and early life environment in the association between changes in family situation and changes in working life is scarce and further studies would be warranted to replicate the findings.

### Implications for research and practice

Our results also point on that the associations between changes in family situation and working life are stronger among men than women. In other words, men’s participation in working life seem more prone to be influenced by the changes in family situation than among women. Although working life participation among women is increasing in most Western countries, access to labour market has remained impeded for women.^20^ One explanation is that, compared with men, women are culturally expected to shoulder the lion share of unpaid care work, such as take household responsibilities and childcare tasks.^20 21^ Combining paid work and family may bring benefits to women,^25 46^ but women may still derive less health benefits from marriage, experience additional stress from combination of work and family and suffer inability to work sustainably.^23^ By contrast, marriage may be a source of both emotional and instrumental support for men since women are more likely to do the household work, which may benefit men’s health and working life participation. As a result, when these potential benefits are interrupted by changes in the family situation such as divorce, men might be more disproportionately affected than women.

### Strengths and weaknesses

This study has several strengths. First, it used a population-based cohort to scrutinise changes in family situation over an extended follow-up period whereas most other studies used cross-sectional data. Bias attributable to loss to follow-up was low as nationwide register data of high quality was used. Many potential confounders were also controlled for. However, despite the comprehensive register data, we lacked information about employment conditions such as working hours, occupation, number of children or any partner information. As these might play a role both in family situation and a sustainable working life, further studies would be needed to confirm these first findings in this study. Another lack in our data was related to the negative effects of family life, for example, for people in situations of domestic abuse or work situations (eg, stress or work pressure, or unhealthy working conditions).^5^ Therefore, such data could be addressed in future studies based on other samples. The large sample also enabled analyses with discordant twin pairs to examine the influence from genetic factors and early life environment. Furthermore, the fixed-effects approach optimises causal inference by removing time-invariant confounding and examined only family situation changes occurring within individuals to estimate the associations of interest, in that way further reducing possible confounder biases. The findings in this study are expected to be generalisable to other populations in developed countries such as Nordic countries with comparable economic and labour market situations, healthcare and social insurance systems, and social norms for marriage and cohabitation as Sweden.

Some limitations should also be noted. First, there was a large proportion of individuals excluded during the follow-up from the analytical sample as the analyses were mainly based on different values and non-missing data of working life. Thus, the sample might be affected by selection bias that might dilute external validity. It should also be highlighted that there might be other potential confounders that are associated with the exposure and outcome as well, such as psychosocial working conditions, or occupation^49^ that we could not include in this study since such data are not at hand. We suggest that these factors should be addressed in future longitudinal studies. Furthermore, data on sickness absence spells <14 days were not available from Social Insurance Agency hence these short absences were included in the assessment of sustainable working life. Although this might be a limitation, it is noteworthy that short absences are common due to various epidemics such as influenza or other viruses. Such absences are essential part of working life, why their role should be investigated in further studies together with other influential factors related to work such as mental and physical demands, management or other organisational factors.

### Conclusions

Single individuals who start to establish family and live with children are more likely to transition from unsustainable to sustainable working life whereas married individuals who experience divorce and those who are not living with children anymore are less likely to transit to sustainable working life. Further, among men, the influence of changes in family situation on changes in working life is more pronounced compared with women. Genetic factors and early life environment also play a role in the associations. Potentially attention should be paid on family formation and remaining with a family to promote sustainable working life and different strategies for women and men should also be considered.

## Data Availability

Data may be obtained from a third party and are not publicly available.

## References

[1] Gaydosh L , Harris KM . Childhood family instability and young adult health. J Health Soc Behav 2018;59:371–90. 10.1177/0022146518785174 29949717 PMC6132243

[2] Seltzer JA . Family change and changing family demography. Demography 2019;56:405–26. 10.1007/s13524-019-00766-6 30838537 PMC6450727

[3] Hendi AS . Proximate sources of change in Trajectories of first marriage in the United States, 1960-2010. Demography 2019;56:835–62. 10.1007/s13524-019-00769-3 30900150 PMC6827978

[4] Gumà J , Cámara AD , Treviño R . The relationship between health and partnership history in adulthood: insights through retrospective information from Europeans aged 50 and over. Eur J Ageing 2015;12:71–9. 10.1007/s10433-014-0316-x 28804347 PMC5549216

[5] Mortelmans D . Chapter 14: causes and consequences of family dissolution in Europe and post-divorce families in. In: Schneider NF , Kreyenfeld M , eds. Research Handbook on the Sociology of the Family. UK and USA. Edward Elgar Publishing Limited, 2021: 232–47.

[6] Blomgren J , Martikainen P , Grundy E , et al . Marital history 1971-91 and mortality 1991-2004 in England & Wales and Finland. J Epidemiol Community Health 2012;66:30–6. 10.1136/jech.2010.110635 20924052

[7] Ahs AMH , Westerling R . Mortality in relation to employment status during different levels of unemployment. Scand J Public Health 2006;34:159–67. 10.1080/14034940510032374 16581708

[8] Guner N , Kulikova Y , Llull J . Marriage and health: selection, protection, and Assortative mating. Eur Econ Rev 2018;104:138–66. 10.1016/j.euroecorev.2018.02.005 33132405 PMC7597938

[9] Eurostat . Marriage and divorce statistics 2023, Available: https://ec.europa.eu/eurostat/statistics-explained/index.php?title=Marriage_and_divorce_statistics

[10] Eurostat . Archive:Marriages and births in Sweden 2018, Available: https://ec.europa.eu/eurostat/statistics-explained/index.php?title=Archive:Marriages_and_births_in_Sweden&oldid=400409

[11] Artazcoz L , Borrell C , Benach J , et al . Women, family demands and health: the importance of employment status and socio-economic position. Soc Sci Med 2004;59:263–74. 10.1016/j.socscimed.2003.10.029 15110418

[12] Laaksonen M , Mastekaasa A , Martikainen P , et al . Gender differences in sickness absence--the contribution of occupation and workplace. Scand J Work Environ Health 2010;36:394–403. 10.5271/sjweh.2909 20213051

[13] Pietiläinen O , Laaksonen M , Rahkonen O , et al . Self-rated health as a Predictor of disability retirement--the contribution of ill-health and working conditions. PLoS One 2011;6:e25004. 10.1371/journal.pone.0025004 21949830 PMC3176797

[14] Heyne S , Voßemer J . Unemployment, and subjective well-being: Why do women suffer less from unemployment than men Eur Sociol Rev 2023;39:301–16. 10.1093/esr/jcac030

[15] Gedikli C , Miraglia M , Connolly S , et al . The relationship between unemployment and wellbeing: an updated meta-analysis of longitudinal evidence. Europ J Work Organizat Psychol 2023;32:128–44. 10.1080/1359432X.2022.2106855

[16] Van Hedel K , Van Lenthe FJ , Avendano M , et al . Marital status, labour force activity and mortality: a study in the USA and six European countries. Scand J Public Health 2015;43:469–80. 10.1177/1403494815578947 25868643 PMC4673396

[17] Caumette E , Vaz-Luis I , Pinto S , et al . The challenge of return to work after breast cancer: the role of family situation. Curr Oncol 2021;28:3866–75. 10.3390/curroncol28050330 34677248 PMC8534983

[18] Wang M , Raza A , Narusyte J , et al . Family-related life events as predictors of labour market Marginalization Trajectories: a cohort study of Swedish twins. J Occup Environ Med 2023;65:627–34. 10.1097/JOM.0000000000002869 37143233 PMC10417248

[19] Voss M , Josephson M , Stark S , et al . The influence of household work and of having children on sickness absence among publicly employed women in Sweden. Scand J Public Health 2008;36:564–72. 10.1177/1403494807088459 18775812

[20] Wood J , Neels K , Wachter D , et al . Family formation and labour force participation: maternal employment and educational differentials in Europe. Population (English Edition, 2002-) 2016;71:53–81. 10.3917/popu.1601.0053

[21] Goldscheider F , Bernhardt E , Lappegård T . The gender revolution: A framework for understanding changing family and demographic behavior. Population & Development Rev 2015;41:207–39. 10.1111/j.1728-4457.2015.00045.x

[22] Meyer T , Pfau-Effinger B . n.d. Gender arrangements and pension systems in Britain and Germany: tracing change over five decades. Int J Ageing Later Life 1:67–110. 10.3384/ijal.1652-8670.061267

[23] Floderus B , Hagman M , Aronsson G , et al . Medically certified sickness absence with insurance benefits in women with and without children. Eur J Public Health 2012;22:85–92. 10.1093/eurpub/ckr028 21450840 PMC3265750

[24] Bratberg E , Dahl SÅ , Risa AE . The double burden’: do combinations of career and family obligations increase sickness absence among women Eur Sociol Rev 2002;18:233–49. 10.1093/esr/18.2.233

[25] Voss M , Floderus B , Diderichsen F . How do job characteristics, family situation, domestic work, and lifestyle factors relate to sickness absence? A study based on Sweden post. J Occup Environ Med 2004;46:1134–43. 10.1097/01.jom.0000145433.65697.8d 15534500

[26] Scruggs LA , Ramalho Tafoya G . Fifty years of welfare state generosity. Soc Policy Adm 2022;56:791–807. 10.1111/spol.12804

[27] Lynch J , Myrskylä M . Always the third rail. Comparative Political Studies 2009;42:1068–97. 10.1177/0010414009331722

[28] SCB . Vanligast att unga och äldre jobbar få timmar 2017, Available: https://www.scb.se/hitta-statistik/artiklar/2017/Vanligast-att-unga-och-aldre-jobbar-fa-timmar

[29] Narusyte J , Ropponen A , Silventoinen K , et al . Genetic liability to disability pension in women and men: a prospective population-based twin study. PLoS One 2011;6:e23143. 10.1371/journal.pone.0023143 21850258 PMC3151284

[30] Kendler KS , Baker JH . Genetic influences on measures of the environment: a systematic review. Psychol Med 2007;37:615–26. 10.1017/S0033291706009524 17176502

[31] Svedberg P , Ropponen A , Alexanderson K , et al . Genetic susceptibility to sickness absence is similar among women and men: findings from a Swedish twin cohort. Twin Res Hum Genet 2012;15:642–8. 10.1017/thg.2012.47 22931554

[32] Carlin JB , Gurrin LC , Sterne JA , et al . Regression models for twin studies: a critical review. Int J Epidemiol 2005;34:1089–99. 10.1093/ije/dyi153 16087687

[33] Ropponen A , Josefsson P , Böckerman P , et al . Sustainable working life patterns in a Swedish twin cohort: age-related sequences of sickness absence, disability pension, unemployment, and premature death during working life. Int J Environ Res Public Health 2022;19:10549. 10.3390/ijerph191710549 36078264 PMC9517844

[34] Ropponen A , Narusyte J , Wang M , et al . Genetic and environmental contributions to individual differences in sustainable working life-A Swedish twin cohort study. PLoS One 2023;18:e0289074. 10.1371/journal.pone.0289074 37498854 PMC10374081

[35] Ludvigsson JF , Svedberg P , Olén O , et al . The longitudinal integrated database for health insurance and labour market studies (LISA) and its use in medical research. Eur J Epidemiol 2019;34:423–37. 10.1007/s10654-019-00511-8 30929112 PMC6451717

[36] Parliament E . Social and Labour Market Policy in Sweden. Report No.: W 13A. Geneva: European Parliament, 1998.

[37] Ropponen A , Wang M , Alaie I , et al . Concurrent Trajectories of residential region in relation to a sustainable working life among Swedish twins. Eur J Public Health 2023;33:596–600. 10.1093/eurpub/ckad053 37029917 PMC10393480

[38] Ropponen A , Wang M , Raza A , et al . Night work and sustainable working life-A prospective trajectory analysis of Swedish twins. Int J Environ Res Public Health 2022;19:10857. 10.3390/ijerph191710857 36078570 PMC9518065

[39] Pforr K . Detailed description of the implementation the Multinomial Logit model with fixed effects. Social Science Open Access Repository 2017.

[40] Allison PD . Fixed Effects Regression Methods for Longitudinal Data Using SAS. SAS Institute, 2005.

[41] Kujala UM , Kaprio J , Koskenvuo M . Modifiable risk factors as predictors of all-cause mortality: the roles of Genetics and childhood environment. Am J Epidemiol 2002;156:985–93. 10.1093/aje/kwf151 12446254

[42] Stammann A , Heiß F , McFadden D . Estimating fixed effects Logit models with large panel data Wirtschaft, Kiel und Hamburg: ZBW - Deutsche Zentralbibliothek Für Wirtschaftswissenschaften, Leibniz-Informationszentrum. contract no.: G01-V3. 2016.

[43] Grundy EMD , Tomassini C . Marital history, health and mortality among older men and women in England and Wales. BMC Public Health 2010;10:554. 10.1186/1471-2458-10-554 20843303 PMC2954998

[44] Dupre ME , Beck AN , Meadows SO . Marital Trajectories and mortality among US adults. Am J Epidemiol 2009;170:546–55. 10.1093/aje/kwp194 19584130 PMC2732990

[45] Rendall MS , Weden MM , Favreault MM , et al . The protective effect of marriage for survival: a review and update. Demography 2011;48:481–506. 10.1007/s13524-011-0032-5 21526396

[46] Kostiainen E , Martelin T , Kestilä L , et al . Employee, partner, and mother:woman’s three roles and their implications for Healthmplications for health. J Fam Issues 2009;30:1122–50. 10.1177/0192513X08329597

[47] Hendriks SM , Spijker J , Licht CMM , et al . Long-term work disability and absenteeism in anxiety and depressive disorders. J Affect Disord 2015;178:121–30. 10.1016/j.jad.2015.03.004 25805404

[48] Kärkkäinen S , Silventoinen K , Svedberg P , et al . Life events as predictors for disability pension due to musculoskeletal diagnoses: a cohort study of Finnish twins. Int Arch Occup Environ Health 2020;93:469–78. 10.1007/s00420-019-01505-5 31828421 PMC7118032

[49] Ropponen A , Wang M , Farrants K , et al . Psychosocial working conditions and subsequent sickness absence-effects of pain and common mental disorders in a population-based Swedish twin sample. J Occup Environ Med 2022;64:451–7. 10.1097/JOM.0000000000002501 35121688 PMC9275835

