# Supplementary material for: Changes in family situation and concurrent changes in working life: a 15-year longitudinal analysis
Source: Fam Med Community Health. 2024 Apr 4;12(2):e002438. doi: 10.1136/fmch-2023-002438 (PMC11002427; doi:10.1136/fmch-2023-002438)
Supplement: Supplementary data [file fmch-2023-002438supp001.pdf]

Supplemental Materials

Supplemental Table S1 Category of changes in family situation between 2005-2020.

**Category of changes in family situation between 2005-2020**
From married without children (reference group) to married with children
From married without children (reference group) to single without children
From married without children (reference group) to single with children
From married with children (reference group) to married without children
From married with children (reference group) to single without children
From married with children (reference group) to single with children
From single without children (reference group) to married without children
From single without children (reference group) to married with children
From single without children (reference group) to single with children
From single with children (reference group) to married without children
From single with children (reference group) to married with children
From single with children (reference group) to single without children

Supplemental Table S2 Ratios of average partial effects (APER) with 95% confidence intervals (CI) in the whole sample

**Men (whole sample)**			
**Change in family situation**	**Crude**	**Model 1**	**Model 2**
	**APER (95%CI)**	**APER (95%CI)**	**APER (95%CI)**
From married without children to married with children	**1.10 (1.09–1.11)**	**1.08 (1.07–1.09)**	**1.08 (1.07–1.09)**
From married without children to single without children	**0.98 (0.97–0.99)**	**0.96 (0.94–0.97)**	**0.96 (0.95–0.97)**
From married without children to single with children	**1.03 (1.01–1.05)**	1.01 (0.99–1.03)	1.01 (0.99–1.03)
From married with children to married without children	**0.90 (0.89–0.91)**	**0.92 (0.91–0.93)**	**0.92 (0.91–0.93)**
From married with children to single without children	**0.89 (0.88–0.89)**	**0.88 (0.87–0.89)**	**0.89 (0.88–0.89)**
From married with children to single with children	**0.93 (0.91–0.95)**	**0.93 (0.91–0.94)**	**0.93 (0.91–0.95)**
From single without children to married without children	**1.02 (1.01–1.03)**	**1.04 (1.03–1.06)**	**1.04 (1.03–1.05)**
From single without children to married with children	**1.12 (1.11–1.13)**	**1.12 (1.12–1.13)**	**1.12 (1.11–1.13)**
From single without children to single with children	**1.05 (1.03–1.07)**	**1.05 (1.03–1.07)**	**1.05 (1.04–1.07)**
From single with children to married without children	**0.97 (0.95–0.99)**	0.99 (0.97–1.01)	0.99 (0.97–1.01)
From single with children to married with children	**1.07 (1.05–1.09)**	**1.07 (1.05–1.09)**	**1.07 (1.05–1.08)**
From single with children to single without children	**0.95 (0.93–0.97)**	**0.95 (0.93–0.96)**	**0.95 (0.93–0.96)**
**Women (whole sample)**			
From married without children to married with children	**1.05 (1.04–1.05)**	**1.03 (1.02–1.03)**	**1.03 (1.02–1.04)**
From married without children to single without children	**0.98 (0.97–0.99)**	**0.96 (0.95–0.97)**	**0.96 (0.95–0.97)**
From married without children to single with children	**1.00 (0.99–1.01)**	**0.98 (0.97–0.99)**	**0.98 (0.97–0.99)**
From married with children to married without children	**0.95 (0.94–0.96)**	**0.97 (0.96–0.98)**	**0.97 (0.96–0.98)**
From married with children to single without children	**0.93 (0.92–0.94)**	**0.93 (0.92–0.94)**	**0.93 (0.92–0.94)**
From married with children to single with children	**0.96 (0.94–0.97)**	**0.95 (0.94–0.96)**	**0.95 (0.94–0.96)**
From single without children to married without children	**1.02 (1.01–1.03)**	**1.04 (1.03–1.05)**	**1.04 (1.03–1.05)**
From single without children to married with children	**1.07 (1.06–1.08)**	**1.07 (1.06–1.08)**	**1.07 (1.06–1.08)**
From single without children to single with children	**1.03 (1.02–1.03)**	**1.02 (1.01–1.03)**	**1.02 (1.01–1.03)**
From single with children to married without children	**1.00 (0.99–1.01)**	**1.02 (1.01–1.03)**	**1.02 (1.01–1.03)**
From single with children to married with children	**1.04 (1.03–1.05)**	**1.05 (1.04–1.06)**	**1.05 (1.04–1.06)**
From single with children to single without children	**0.97 (0.96–0.98)**	**0.98 (0.97–0.99)**	**0.98 (0.97–0.99)**

Supplemental Table S3 Ratios of average partial effects (APER) with 95% confidence intervals (CI) among the discordant twins

Change in family situation	Conditional all	DZ pairs	MZ pairs
	**APER (95%CI)**	**APER (95%CI)**	**APER (95%CI)**
**Men (discordant twins)**			
From married without children to married with children	1.04 (1.00–1.08)	0.99 (0.93–1.05)	1.02 (0.96–1.10)
From married without children to single without children	**0.91 (0.87–0.95)**	0.95 (0.89–1.02)	**0.86 (0.80–0.92)**
From married without children to single with children	**0.90 (0.83–0.97)**	1.00 (0.90–1.13)	0.88 (0.76–1.01)
From married with children to married without children	0.96 (0.92–1.00)	1.01 (0.95–1.07)	0.98 (0.91–1.05)
From married with children to single without children	**0.87 (0.85–0.90)**	0.96 (0.91–1.01)	**0.84 (0.81–0.87)**
From married with children to single with children	**0.85 (0.80–0.92)**	1.01 (0.92–1.12)	**0.85 (0.75–0.96)**
From single without children to married without children	**1.09 (1.06–1.13)**	1.05 (0.99–1.11)	**1.15 (1.08–1.21)**
From single without children to married with children	**1.14 (1.11–1.17)**	1.04 (0.99–1.09)	**1.18 (1.14–1.23)**
From single without children to single with children	1.00 (0.94–1.06)	1.05 (0.96–1.15)	1.04 (0.95–1.14)
From single with children to married without children	**1.10 (1.03–1.16)**	1.00 (0.89–1.12)	**1.11 (1.01–1.22)**
From single with children to married with children	**1.14 (1.08–1.20)**	0.99 (0.89–1.10)	**1.14 (1.04–1.25)**
From single with children to single without children	1.00 (0.95–1.06)	0.95 (0.86–1.05)	0.96 (0.87–1.06)
**Women (discordant twins)**			
From married without children to married with children	0.98 (0.95–1.01)	**0.94 (0.89–0.99)**	1.01 (0.96–1.06)
From married without children to single without children	**0.88 (0.85–0.91)**	**0.86 (0.81–0.92)**	**0.90 (0.86–0.95)**
From married without children to single with children	**0.90 (0.86–0.94)**	**0.89 (0.82–0.97)**	0.93 (0.88–1.00)
From married with children to married without children	1.02 (0.99–1.05)	**1.06 (1.01–1.11)**	0.99 (0.95–1.04)
From married with children to single without children	**0.90 (0.88–0.92)**	**0.92 (0.88–0.96)**	**0.89 (0.86–0.92)**
From married with children to single with children	**0.92 (0.89–0.95)**	0.95 (0.89–1.01)	**0.92 (0.88–0.97)**
From single without children to married without children	**1.12 (1.09–1.15)**	**1.13 (1.08–1.19)**	**1.09 (1.05–1.14)**
From single without children to married with children	**1.11 (1.08–1.13)**	**1.08 (1.04–1.13)**	**1.11 (1.08–1.15)**
From single without children to single with children	1.02 (1.00–1.05)	1.03 (0.98–1.09)	1.03 (1.00–1.07)
From single with children to married without children	**1.10 (1.06–1.13)**	**1.10 (1.04–1.17)**	**1.06 (1.01–1.12)**
From single with children to married with children	**1.08 (1.05–1.11)**	1.05 (0.99–1.11)	**1.07 (1.03–1.12)**
From single with children to single without children	0.98 (0.95–1.00)	0.97 (0.91–1.03)	0.96 (0.93–1.00)
